# Advancements in Dentistry with Artificial Intelligence: Current Clinical Applications and Future Perspectives

**DOI:** 10.3390/healthcare10112188

**Published:** 2022-10-31

**Authors:** Anum Fatima, Imran Shafi, Hammad Afzal, Isabel De La Torre Díez, Del Rio-Solá M. Lourdes, Jose Breñosa, Julio César Martínez Espinosa, Imran Ashraf

**Affiliations:** 1 National Centre for Robotics, National University of Sciences and Technology (NUST), Islamabad 44000, Pakistan; 2 College of Electrical and Mechanical Engineering, National University of Sciences and Technology (NUST), Islamabad 44000, Pakistan; 3 Military College of Signals (MCS), National University of Sciences and Technology (NUST), Islamabad 44000, Pakistan; 4 Department of Signal Theory and Communications and Telematic Engineering, University of Valladolid, Paseo de Belén 15, 47011 Valladolid, Spain; 5 Department of Vascular Surgery, University Hospital of Valladolid, Paseo de Belén 15, 47011 Valladolid, Spain; 6 Universidad Europea del Atlántico, Isabel Torres 21, 39011 Santander, Spain; 7 Universidad Internacional Iberoamericana, Arecibo, PR 00613, USA; 8 Universidade Internacional do Cuanza, Estrada Nacional 250, Bairro Kaluapanda Cuito- Bié, Angola; 9 Universidad Internacional Iberoamericana, Campeche 24560, Mexico; 10 Fundación Universitaria Internacional de Colombia, Calle 39A #19-18 Bogotá D.C, Colombia; 11 Department of Information and Communication Engineering, Yeungnam University, Gyeongsan 38541, Korea

**Keywords:** dentistry, artificial intelligence, deep learning, clinical applications

## Abstract

Artificial intelligence has been widely used in the field of dentistry in recent years. The present study highlights current advances and limitations in integrating artificial intelligence, machine learning, and deep learning in subfields of dentistry including periodontology, endodontics, orthodontics, restorative dentistry, and oral pathology. This article aims to provide a systematic review of current clinical applications of artificial intelligence within different fields of dentistry. The preferred reporting items for systematic reviews (PRISMA) statement was used as a formal guideline for data collection. Data was obtained from research studies for 2009–2022. The analysis included a total of 55 papers from Google Scholar, IEEE, PubMed, and Scopus databases. Results show that artificial intelligence has the potential to improve dental care, disease diagnosis and prognosis, treatment planning, and risk assessment. Finally, this study highlights the limitations of the analyzed studies and provides future directions to improve dental care.

## 1. Introduction

Artificial intelligence (AI) has been widely utilized in the field of medicine since its conception over 60 years ago [1,2,3,4]. Although the maturity of AI in the field of dentistry has lagged in several subfields such as periodontology, endodontics, orthodontics, restorative dentistry, and oral pathology, there has been a large interest in the past few years as artificial intelligence has become increasingly accessible to researchers. AI has made substantial progress in the diverse disciplines of dentistry including dental disease diagnosis [5], localization [6], classification [7], estimation [8], and assessment of dental disease [9].

On a broader level, AI enables the creation of intelligent machines that can achieve tasks without requiring human intervention. Machine learning (ML) [10] is a subset of AI that utilizes computational algorithms to analyze datasets to make predictions without the need for explicit instructions. Towards a more sophisticated and increasingly independent approach for diagnosis, treatment planning, and risk assessment, there has been increased interest in deep learning (DL) applications [11,12]. To provide expert support to healthcare practitioners, artificial neural networks (ANNs) can be utilized as clinical decision support systems (CDSS) [13]. Moreover, such systems aid dental clinicians in producing improved dental health outcomes [14]. Similarly, fuzzy logic (FL)-based CDSS also provides effective means for dealing with uncertainties in the decision-making process [15,16].

### 1.1. Background

The related background of ANNs, machine learning, deep learning, CDSS, and Fuzzy logic is discussed in the below subsections.

#### 1.1.1. Artificial Neural Networks

ANNs evolved after McCulloch and Pitts created a model in 1943 based on many simple processing units, such as neurons connected using weighted links. In 1958, Rosenbolt published neurodynamic principles containing research ideas related to brain modeling. Later, in the 1980s, researchers developed algorithms based on the idea of backpropagation to be applied in the medical field [1].

ANNs comprise data processing mechanisms inspired by the analytical processes of the human brain. The ANNs are used extensively to solve complex real-world problems [2]. Due to ANN’s remarkable learning, generalization, inherent contextual information processing, and fault and noise tolerance capabilities, they provide exciting alternatives and have many applications including medicinal science such as disease diagnosis, image and data analysis, and biomedical identification [3,4].

#### 1.1.2. Machine Learning

ML is the scientific study of algorithms and statistical models [10] used for a vast array of processing tasks without requiring prior knowledge or hand-crafted rules. Recent years have witnessed the widespread of ML due to its superior performance for various healthcare applications such as dentistry. ML algorithms fall into two learning types: supervised and unsupervised. The amount of data generated by healthcare service providers is huge, making the data analysis process cumbersome. ML helps in effectively analyzing the data and gaining actionable insights. Additionally, different dentistry applications can benefit from ML techniques, including disease diagnosis, prognosis, treatment, and automating the clinical workflow. Moreover, ML for clinical applications has great potential to transform traditional healthcare service delivery [17].

#### 1.1.3. Deep Learning

Recent years have seen a surge of interest in the DL field, a subfield of ML, as it allows machines to mimic human intelligence in increasingly independent and sophisticated ways [11,12]. DL uses multiple layers of non-linear units to analyze and extract useful knowledge from huge amounts of data. The extracted knowledge is then used to produce state-of-the-art prediction results. The neural network architectures used in DL provide the capability to perform automatic and accurate detection in healthcare. Based on the study, DL has enormous potential to bring genuinely impactful applications to the field of dentistry.

#### 1.1.4. ANNs as Clinical Decision Support Systems

AI application technology is progressing remarkably in the field of dentistry; clinical decision support systems are one example in this context. CDSS provides expert support to a health practitioner [13]. Moreover, these systems have the capability of solving problems that are too complex to be solved by using conventional methods [14]. Moreover, CDSS provides valuable information to dental practitioners that aids in producing faster and superior dental health outcomes.

#### 1.1.5. Fuzzy Logic

In the past decade, fuzzy logic has proven to be a powerful tool for decision-making systems [15]. The fuzzy set theory derives from the fact that the natural classes and concepts are vague. This makes fuzzy logic quite suitable for complex systems as they can summarize from massive information inputs with increased effectiveness and tolerance to imprecisions [16]. The study provides brief descriptions of the key contributions made by fuzzy technology in the field of dentistry.

### 1.2. Motivation and Contributions

Several surveys addressing the use of AI in dentistry have been published in recent years. Ossowoska et al. [18] discuss the possibilities of using neural networks in different fields of dentistry including restorative dentistry, endodontics, orthodontics, dental surgery, and periodontology. The study indicates that artificial intelligence has developed rapidly in recent years and has the potential to be applied in routine dentistry. However, the study lacks discussion related to the limitations of the included studies. This study aims to outline the clinical support decision systems developed in recent years possess the potential to be employed in different fields of dentistry. We also discuss the limitations and formulate future directions of such approaches to contribute to improving the current state of clinical dental practice. The contributions of this study are provided below

- Identify the development of AI applications that are employed widely in dentistry for different diagnostic tasks such as disease diagnosis, risk assessment, treatment planning, and prognosis and evaluate their diagnostic performance,
- Outline the limitations of the neural networks employed for different diagnostic tasks including disease diagnosis, landmark detection, risk assessment, treatment planning, and prognosis,
- Provide future directions which may have a positive and stimulating impact on the future application of artificial intelligence-based techniques in the field of dentistry.

### 1.3. Article Organization

The rest of this survey is organized as follows. Survey methodology is explained in Section 2. Applications of AI approaches are discussed in Section 3 while the limitations and future directions are provided in Section 4. In the end, the survey is concluded in Section 5.

## 2. Methodology

For this article, the preferred reporting items for systematic reviews (PRISMA) guidelines are followed. PRISMA provides a standard peer-accepted methodology consisting of a guideline checklist to ensure the quality assurance of the work’s replicability. This section defines the article selection criteria, data sources, and the search strategy for data extraction and analysis procedure.

### 2.1. Research Questions

Following are the research questions formulated to analyze the relevant studies:

RQ1. What are the main fields in dentistry in which AI is employed?
RQ2. What are data modalities and features used for diagnosing dental diseases?
RQ3. Which AI techniques (including subfields) are utilized for disease diagnosis in different fields of dentistry?
RQ4. Which outcome measures are used for the performance assessment of models?

Furthermore, the research question was formatted using population, intervention, comparison, and outcome (PICO):

- Population: Dental imagery related to radiographs (bitewing, periapical, occlusal, panoramic, cephalograms, cone-beam computed tomography (CBCT)), digital photographs, high-frequency ultrasound images, 3D CBCT images, domain, near-infrared light transillumination (NILT) images, extraoral/intraoral mold images, orthodontic scans, magnetic resonance imaging, water’s view radiographs, hyperspectral images and textual data including electronic health records.
- Intervention: Artificial intelligence, machine learning, deep learning-based models for diagnosis, detection, classification, segmentation, risk assessment, treatment planning, and prognosis.
- Comparison: Reference standard, expert opinions.
- Outcome: Measurable and predictive outcomes that include accuracy, specificity, sensitivity, F1-score, intersection over union (IoU), dice co-efficient, regression co-efficient receiver operating characteristic curve (ROC), and area under the curve (AUC), successful detection rate (SDR).

### 2.2. Data Sources and Search Strategies

Different electronic databases were systematically searched including Google Scholar, Institute of Electrical and Electronics Engineers (IEEE) Xplore, PubMed, and Scopus. The search terms were broadened to identify as many eligible studies as possible. The search key strategy adopted for each database is given in Table 1.

### 2.3. Selection of Studies

**Figure 1:**
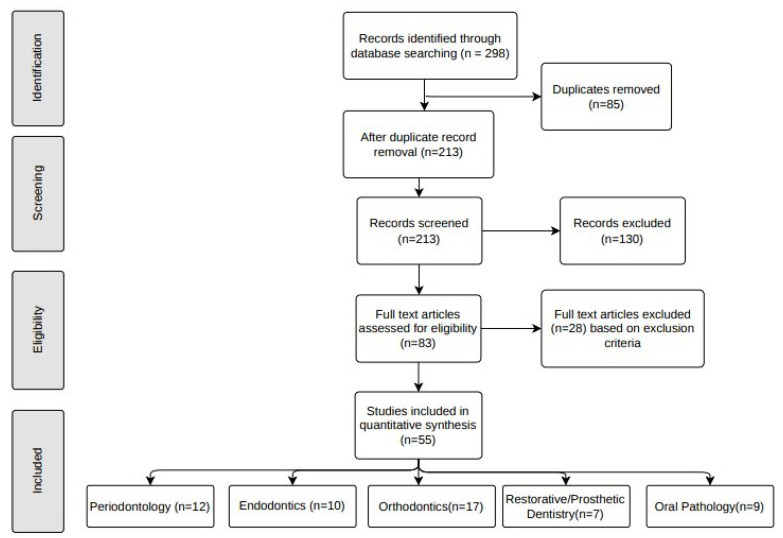
Relevant data about studies included for synthesis.

After an initial literature search was conducted, the title and abstract were screened for each study. Potentially relevant studies were further assessed for eligibility. Detailed information about the selection process is depicted in Figure 1.

For risk assessment of bias for each study, sampling and measurement bias was assessed. The articles are identified based on the library searches. A total of 298 studies (Google Scholar, n = 128, PubMed, n = 66, IEEE Xplore, n = 58, and Scopus, n = 46) were identified via the initial search process. After examining the abstracts and titles of the identified studies, duplicates (n = 85) were excluded. The remaining 213 studies were screened, out of which 130 articles without full-text availability were eliminated. The full-text articles were assessed for eligibility and based on the exclusion criteria 28 articles were excluded. Following these procedure, 55 eligible studies (periodontology, n = 12, endodontics, n = 10, orthodontics, n = 17, restorative/prosthetic dentistry, n = 7, oral pathology, n = 9) were included in this review.

### 2.4. Eligibility Criteria

The following inclusion criteria were used in this study

1. Publications between 2009 and 2022 related to the application of artificial neural networks, machine learning, deep learning, fuzzy logic, expert systems, and clinical support decision systems (CDSS) in dentistry.
2. Studies in the English language and with full-text availability.
3. Published in a scholarly peer-reviewed journal.
4. Publications truly implementing AI including subfields in the context of dental topics.

The studies were excluded from the review if

1. Not published in a peer-reviewed journal.
2. Unpublished thesis or dissertation studies.
3. Studies that did not use artificial intelligence, review articles, and letter to the editor.

### 2.5. Risk Assessment

Each study was evaluated by the reviewers. To further minimize the risk of bias, studies dealing with AI implementation were included. Initial disagreements among the reviewers were noted while assessing the selected studies.

## 3. Applications of Artificial Intelligence in Dentistry

**Figure 2:**
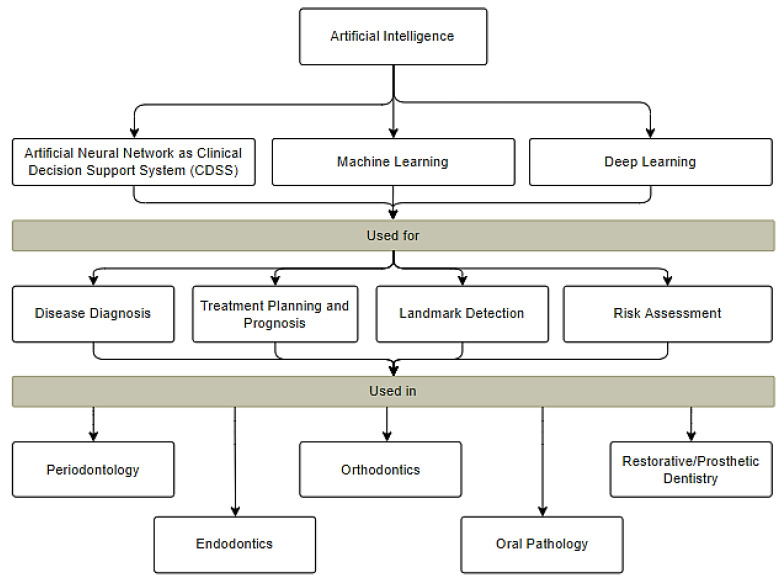
Applications of AI in different subfields of dentistry.

AI has made substantial progress in the diverse disciplines of medicine, specifically in the field of dentistry for diagnosis [5], localization [6], classification [7], estimation [8], and assessment of dental disease. With the recent rapid development of AI technologies designed for dental practitioners, dental clinicians make precise diagnoses and provide accurate recommendations. Figure 2 shows the areas within dentistry where AI can be used followed by a detailed description of AI applications in these fields.

The progressive development of AI in dentistry will benefit both researchers and clinicians in integrating different fields of knowledge to improve patient care. There are different sub-disciplines of AI that are being used to provide precise, cost-effective, and user-friendly solutions to facilitate dentists and clinicians including ANNs and ANNs as CDSS, ML, and DL. The diagnostic activities performed using different input features in dentistry are shown in Table 2.

### 3.1. AI in Periodontology

In periodontology, AI has been used extensively to explore, understand and develop periodontal applications including periodontal bone loss detection, diagnosing gingivitis inflammation, and assessment of connective tissues and other periodontal caries. Table 3 shows different studies that are introduced in periodontology. These studies indicate that AI-assisted systems can be used in clinical practices to strengthen and improve dental treatment outcomes in a more sophisticated manner.

Moreover, to minimize errors in diagnosis, authors have proposed methods involving machine learning techniques. Li et al. proposed a plaque segmentation method based on a convolutional neural network (CNN) using oral endoscopic images. The results provided performance superior to that of dentists with an accuracy of 86.42% [29]. An extreme machine learning method based on contrast limited adaptive histogram and gray-level covariance matrix was evaluated by Li and fellow authors, using digital photographs. The model provided an accuracy of 74% for gingivitis identification using a small dataset [30]. For localization of alveolar bone loss, Lin et al. evaluated a level segmentation method based on a support vector machine (SVM), k nearest neighbor (KNN), and Bayesian classifier. The model was able to localize alveolar bone loss with high classification effectiveness [6].

DL-based methods have gained immense popularity in recent years. Several authors have evaluated methods for bone loss detection in intraoral radiographs, Lee et al. proposed a VGG-based neural network for diagnosing periodontal bone loss using 1740 periapical radiographs. The model achieves an accuracy of 99% and AUC of 98% outperforming the performance of three dentists [41]. Another model using deep-feed forward CNN was evaluated by Krois and fellow authors using panoramic radiographs. The model showed discrimination ability similar to that of three examiners [5]. Kim et al. and Lee et al. suggested the use of transfer learning to improve the performance of bone loss and odontogenic cyst lesion detection using panoramic radiographs. The model was useful in tooth numbering with performance superior to that of dental clinicians [44,63]. For the classification of regions based on periodontal bone destruction, Moran et al. demonstrated a ResNet model achieving an accuracy of 82% using 467 periapical radiographs [7]. To automate the process of detecting bone lesions and detecting correct shapes, Khan et al. presented a disease segmentation method based on U-Net architecture. The model was able to detect the presence and shape of caries with performance higher than three experts [44]. Another anatomically constrained dense U-Net method was proposed by Zheng et al. for bone lesion identification. Using cone beam computed tomography (CBCT) images, the model was able to detect the correct shape of the bone and lesion [8]. Duong et al. proposed a U-Net-based network for alveolar bone delineation using high-frequency ultrasound images yielding performance higher than three experts [45]. Using ResNet34 as an encoder with U-Net, Nguyen et al. assessed 1100 intraoral images for alveolar bone segmentation. The model was able to identify alveolar bone with a dice coefficient of 85.3% [46]. For periodontitis detection, Li et al. utilized Mask R-CNN with a novel calibration method. Using panoramic radiographs, the model diagnosed the severity degree of periodontitis with an accuracy of 82% outperforming that of a junior dentist [64].

In dentistry, the interpretation of data and carrying out proper diagnosis are crucial. However, medical decision-making is cumbersome for doctors in a time-compressed environment. Thus, an intelligent tool is required to assist doctors in making accurate decisions. These systems come under the category of CDSS. ANNs have been used as CDSS for diagnosis, classification, and assessment. Several ANN architectures have been employed to assist doctors in making accurate decisions in periodontology. Recently, Geetha et al. proposed a back propagation neural network for tooth decay detection. The model achieved an accuracy of 97.1% and a false positive rate of 2.8% using intraoral radiographs. The study indicates that ANNs can be employed for precise decay detection compared to traditional dental examination methods [19]. Papantonopoulos et al. evaluated multilayer perceptron ANN for bone loss assessment on medical health records. The model provided effective periodontitis classification with an accuracy of 98.1% [23]. Using 230 textual subjects, Shankarapillai et al. proposed a multilayer feed-forward propagation network for effective periodontitis risk prediction [9].

### 3.2. AI in Endodontics

Endodontics is the branch of dentistry concerned with diseases of the dental pulp and tissues surrounding the root or tooth. AI has gained immense popularity in aiding treatment planning in endodontics. AI with different models can help dentists to diagnose, and manage endodontic problems with encouraging performance and ensuring enhanced and precise patient care. The review is focused on extracting and analyzing AI-based approaches for disease diagnosis and treatment planning. The summary of relevant studies for AI application in endodontics to provide better dental treatment outcomes is shown in Table 4.

Ghaedi et al. presented a circular Hough transform-based segmentation method to detect and score caries lesions in intraoral occlusal tooth surface images. The model yielded an accuracy of 86.3% in detecting and scoring caries lesions [31]. A random forest-based machine learning algorithm has been proposed by Berdouses et al., evaluated on colored images, the model achieved an accuracy of 80% higher compared to two pediatric dentists [32].

Different DL models have also been employed in endodontics for disease detection, classification, and segmentation. Pauwels et al. evaluated a transfer learning-based CNN using intraoral radiographs to detect periapical lesions. The model achieved superior performance compared to three radiologists [47]. Fukuda et al. performed vertical root fracture detection using DetectNet on 300 panoramic radiographs. The model was found to be useful and provided better performance for fracture detection compared to three observers [49]. Orhan et al. proposed the U-Net model to diagnose periapical lesions using 3D CBCT images. The model was able to detect periapical lesions with 92.8% reliability [48]. A seven-layer feed-forward network was presented by Ekert et al. for diagnosing apical lesions using panoramic radiographs. Compared to six independent and experienced dentists, the model was able to detect lesions with an AUC of 85% [50]. Bayraktar and Aryan presented an interproximal caries detection method based on you look only once (YOLO) pre-trained using DarkNet-53 using bitewing radiographs. The authors achieved an accuracy of 94.59% higher compared to two experienced dentists [51]. For classification and assessment of distal roots, Hiraiwa et al. evaluated AlexNet achieving a detection performance of 87.4% superior to that of dental radiologists in differentiating whether the distal root was single or with an extra root [52]. Symmetric autoencoder with skip connections similar to U-Net and the encoding path was presented by Casalegno et al. to detect proximal occlusal caries using 217 grayscale near-infrared transilluminations (NILT) images. The model achieved higher throughput in detecting occlusal and proximal lesions compared to two dentists [53]. Kositbowornchai et al. presented a probabilistic neural network to be used as CDSS for diagnosing vertical root features. The model achieved an accuracy of 95.7% on intraoral digital radiographs [20].

### 3.3. AI in Orthodontics

Recent years have witnessed the immense popularity of AI in orthodontics due to its ability to make the diagnostic process more accurate and efficient. Orthodontic treatments are usually long procedures which is why more efficient solutions are required for effective and efficient planning. AI-based knowledge has the potential to automate disease diagnosis and treatment prognosis processes to help dental clinicians in making decisions more accurately and efficiently in a time-constrained environment. AI applications can further help in preventing human errors through their ability to learn and make automotive decisions. Different studies have been explored that incorporate the use of AI for orthodontic disease diagnosis and treatment planning which are shown in Table 5.

Several authors have adopted machine learning techniques for disease diagnosis and treatment planning in the field of orthodontics. Chen et al. proposed a machine learning algorithm based on a multisource integration framework for assessing maxillary structures using CBCT images. The model achieved encouraging performance with a dice ratio of 0.80 in assessing maxillary structure variation in unilateral canine impaction [33]. An SVM-based algorithm was evaluated by Nino-Sandoval to classify skeletal patterns using craniomaxillary variables using cephalograms. The model achieved an accuracy of 74.5% in analyzing sagittal skeletal patterns [34]. Yu et al. investigated support vector regression (SVR) to rate facial attractiveness from most attractive to least attractive using colored photographs. It was found that the model can be used in finding a close correlation with facial attractiveness from orthodontic photographs with an accuracy of 71.8% [35]. Different studies explored the use of ML in treatment planning. Riri et al. proposed a tree-based classification method to identify facial and skin molds using intraoral and extraoral mold images. The model yielded an accuracy of 94.28% [37]. Suhail et al. proposed a random forest ensemble method to automate the extraction and treatment planning process. Based on patients’ medical health records, the model was able to make decisions for teeth extraction with an accuracy of 94.4% [38].

Literature addressed landmark detection problems as well using DL techniques. Song et al. evaluated a ResNet-50 model pre-trained using transfer learning on 400 cephalograms to detect cephalometric landmarks. The model achieved a superior SDR for two test cases in detecting 19 landmarks compared to two experienced doctors [60]. Kim et al. presented a two-stage deep neural network (DNN) with a stacked hourglass network for landmark detection using 2075 cephalograms followed by a web application. The model provided SDR of 82.92% and 84.53% for dataset 1 and dataset 2, respectively [65]. Gilmour et al. proposed pre-trained ResNet-50 with foveated pyramid attention algorithm achieving an SDR of 88.32% and 77.05% for dataset 1 and dataset 2, respectively [66]. Park et al. performed landmark detection using YOLOv3 on 1311 cephalograms. The model was effective in identifying 80 landmarks with 5% higher accuracy compared to top benchmarks. Moreover, attention-based networks have also been explored actively in landmark detection [61]. Zheng et al. explored attention guided deep regression model through a two-stage U-Net using 300 cephalograms. The model achieved an SDR of 86.74% which was higher compared to that of two experienced doctors [8]. Different deep learning-based methods have been employed for disease diagnosis. Makeremi et al. proposed a customized CNN to determine cervical vertebra maturation degree using cephalograms. The model achieved an accuracy of 95% helpful in determining the degree of maturation of CVM and has the potential to be implemented in real-world scenarios [68]. Yu et al. presented modified DenseNet pre-trained with ImageNet weights using lateral cephalograms. The model yielded an accuracy of 95.70% superior to that of five orthodontic specialists [69]. Deep learning for treatment planning has also gained attention in recent years. Lee et al. proposed a modified AlexNet for differential diagnosis in orthodontic surgery achieving an accuracy of 91.9% [62]. The study indicated that deep CNNs-based models can be applied for differential diagnosis in orthodontic surgery.

Different studies have explored ANNs as CDSS in orthodontics. Amasya et al. proposed ANN to diagnose cervical vertebra maturation degree using cephalograms. The model achieved an accuracy of 86.93% in CVM staging and cervical vertebral morphology classification [21]. Kok et al. proposed an ANN-based network to classify cervical vertebrae stages. It was found that ANN provided the most stable results with a 2.17 average rank on hand-wrist radiographs compared to an orthodontist [70]. Another ANN-based method was evaluated by Budiman using orthodontic scans to detect the shape of the arch form. The model achieved an accuracy of 76.32% [22]. In terms of treatment planning and prognosis, different studies explored the use of ANNs. Choi et al. proposed an ANN-based method for diagnosing and making surgery type and extraction decisions effectively [24].

### 3.4. AI in Restorative Dentistry and Prosthodontics

The number of studies exploring the use of AI in restorative dentistry has increased considerably in recent years. Different studies explored the use of AI in assisting the diagnosis of caries, and vertical tooth fracture, predicting restoration failure, and planning treatment. Table 6 shows the relevant studies for AI application in restorative dentistry to provide better dental treatment outcomes.

The usefulness of AI in restorative dentistry has been investigated by several researchers. Lee et al. proposed a machine learning method based on a decision tree to determine tooth prognosis for effective treatment planning using clinical cases. The model achieved an accuracy of 84.1% [39]. A cubic SVM-based algorithm was proposed by Abdalla-Aslan et al. using panoramic radiographs. The model has the potential to detect and classify dental restorations to promote patients’ health [40].

Lee and Jeong proposed a fine-tuned and pre-trained deep CNN to diagnose dental implants using panoramic radiographs. The model yielded an AUC of 97.1% for the identification and classification of dental implants with performance similar to that of periodontists [54]. A deep CNN pre-trained with ImageNet weights was proposed by Takahashi et al. to diagnose partially edentulous arches achieving an accuracy of 99.5% for maxillar arches and 99.7% for mandible arches on oral photographs. The study showed that deep learning can be used effectively in designing removable partial dentures [71]. To preserve the teeth boundary, Xu et al. presented a two hierarchical CNN for segmentation of upper and lower teeth using 3D dental model images [72].

In terms of treatment planning and prognosis, ANNs have been utilized in different studies. Cui et al. proposed a triple classification algorithm based on extreme gradient boost (XGBoost). Using electronic health records, the model yielded an accuracy above 90% superior to that of prosthodontists in predicting tooth extracting therapy [25]. Another feed-forward ANN was presented by Javed et al. to identify occlusal dentinal caries lesions. The study proposed an iOS app for the meticulous prediction of caries using clinical cases [26].

### 3.5. AI in Oral Pathology

The relevant studies utilizing AI for disease diagnosis and prognosis are given in Table 7. Orhan et al. proposed a machine learning algorithm based on KNN and random forest using magnetic resonance images to diagnose temporomandibular disorders. The model achieved an accuracy of 77% in identifying condylar changes and 74% for disc displacement [36]. Hung et al. presented a three-step CNN-based on V-Net and SVR to identify maxillary sinusitis using CBCT images. The model yielded an AUC of 92% for mucosal thickening and 84% for mucous retention cyst identification [55].

In a similar fashion, Kuwana et al. evaluated DetectNet on panoramic radiographs to detect maxillary sinus lesions with an accuracy of 90 to 91% for maxillary sinusitis and 97 to 100% for maxillary sinus cysts [56]. Moreover, a deep CNN (ResNet) was presented by Choi et al. to diagnose temporomandibular joint disorders (TMJ) osteoarthritis. The model yielded an accuracy of 78% similar to that of Oral and maxillofacial radiologists (OMFR) [58].

Kim et al. investigated the performance of CNN on water’s view radiographs to identify maxillary sinusitis. The model achieved significantly higher diagnostic performance compared to that of five radiologists [42]. Murata et al. evaluated the deep learning model AlexNet to diagnose maxillary sinusitis on panoramic radiographs. The model showed diagnostic performance similar to the radiologists and superior to the resident dentists [57]. Jeyaraj et al. proposed a partitioned CNN based on GoogleNet and InceptionV3 for diagnosing oral cancer using hyperspectral images. The model achieved an accuracy of 91.4% and 94.5% for benign and malign tissue respectively [59].

Clinical support decision systems have also been used in oral pathology. Iwasaki et al. proposed a Bayesian belief network (BNN) using magnetic resonance images to diagnose TMJ disorders. The model achieved an accuracy of 99% and can be used to determine the progression of TMD in terms of bone changes, disc displacement, and bony space by dental clinicians [27]. Moreover, a backpropagation ANN has been proposed by Bas et al. to identify clinical symptoms of TMJ disorders. It was found that the model can help diagnose the preliminary subtypes of TMJ and can be useful in the decision-making process [28].

## 4. Limitations and Future Direction

This study confirms the growing presence of AI in dentistry and highlights the performance of AI-based techniques as clinical support decision systems. Although AI techniques have been integrated increasingly into different subfields of dentistry, the current evidence of AI in clinical practice is still very limited. This section discusses the limitations and provides some future directions which may have a positive and stimulating impact on the future application of AI-based techniques in the field of dentistry.

### 4.1. Limitations

Although AI techniques have been employed increasingly within the field of dentistry, certain limitations need to be addressed to improve the performance, reliability, and generalizability of AI-based models. These limitations are discussed here

- Computational capability: AI-based techniques require a significant amount of parallel processing power to keep up with the demands. There are certain limitations of computational resources such as RAM and GPU cycles that are required to support such approaches.
- Reliability: It is essential in a clinical setting and involves ethical fairness, the accuracy of the system, and patients’ trust/acceptance of such systems. Limited reliability causes spurious consequences.
- Generalizability: Issues in generalizability are a dominant concern for clinical guidelines, and a precise level of generalizability is required for the application of AI-based models in clinical practices and ensuring that it performs well prospectively.
- Class imbalance: The difference in the number of samples representing each class within the data is referred to as class imbalance. The lack of high-quality and labeled datasets limits the deployment of robust and accurate AI-based models.
- Overfitting: Due to the high complexity in terms of parameters of deep learning models, the models are more likely to overfit with the training data, thus affecting the generalizability of the results.

The limitations of each relevant study covered in this review are given in Table 8 and Table 9. One of the most prevailing limitation in majority of the studies involving application of AI in subfields of dentistry is dataset limitation in terms of size for disease diagnosis [5,19,31,42,44,45,51,56,57,58,59,71], for treatment planning and prognosis [29,37,62], and for landmark detection [64]. Other limitations include relying on the reliability of examiners for accurate diagnosis [41], less flexibility to capture nonlinearities in data [23], limited practical use [54], poor performance on images that are not evenly illuminated [6], limited class discrimination [6], lack in terms of generalizability [29], lack in terms of performance [9,28,60,61,62,64,65,66,68,69], and computational capabilities [67].

### 4.2. Future Directions

Keeping in view the current limitations of studies employing AI for disease diagnosis, treatment planning and prognosis, landmark detection, and risk assessment, the following are a few research directions that can help in elevating the deep learning model’s performance, reliability, and generalizability:

#### 4.2.1. Data Augmentation

Class-imbalanced datasets are biased towards majority classes, hence the chances for misclassification are often higher. However, different techniques can be employed to deal with the class imbalance and improve the performance of deep learning models. Certain techniques can be utilized to minimize class imbalance, such as data augmentation. To extract the increased amount of information from medical images, non-linear transformations can be applied. Generative adversarial networks (GANs) can prove to be a good alternative for creating similar images using non-linear transformations inside the network. Particularly, GANs are being used increasingly for medical data augmentation such as on pulmonary computed tomography (CT) images to detect COVID-19 lesions [73] and on small-scale medical image datasets [74]. To get a powerful variation of the generative model that can be employed to overcome the class imbalance in a more sophisticated way, known as balancing generative adversarial networks (BAGANs). It focuses on generating minority-class images of high quality.

#### 4.2.2. Cross Center Training

One of the limitations of deep learning application in dentistry is the uncertainty about the generalizability of the models. Limited generalizability may be related to differences in terms of population characteristics such as dental status, and image characteristics such as differences in data generation protocols involved. Thus, understanding the reasons behind generalizability limitations will help in formulating strategies to mitigate generalizability problems. Further research should be focused on employing cross-center training [75] to increase the generalizability of deep learning-based models.

#### 4.2.3. Robotics in Dentistry

Similar to other fields, dentistry is also moving forward toward a new era of data-driven medicine assisted by robots. Robotic dental assistance [76] has the potential to be applied to different fields including orthodontics, implant dentistry, and prosthodontics. To improve the applicability of AI in dentistry, more flexible systems are needed to reach human-level performance and further improve the reliability of AI-based models in clinical practice.

#### 4.2.4. Virtual Reality and Augmented Reality

For practicing safe and efficient dentistry and gaining simultaneous feedback, virtual reality (VR) can be adopted. To generate clinical information that can be visualized by patients, augmented reality (AR) can be adopted. The use of AR and VR is increasing gradually, and there is a limited number of studies that assessed their use [77]. From providing better visualization to reducing operative time and improving patient consultation and providing promising treatment outcomes, AR and VR have the potential to enable researchers in developing high-quality tools for clinical practice. Clinicians can make use of AR to enable patients to visualize expected outcomes before undergoing the procedure. AR and VR can be employed to provide improved dental education by enhancing students’ learning experiences during pre-clinical training [78].

## 5. Discussion and Conclusion

It is suggested that the AI-based systems developed in recent years possess the potential to be employed in different fields of dentistry to help dental clinicians and practitioners in making precise diagnoses and providing accurate recommendations. However, AI is still unlikely to replace the dentist-patient relationship in the foreseeable future as humane elements are also of utmost importance in decision-making to manage dental care. The AI technologies are intended to support dental clinicians in reducing misdiagnosis and work synergistically with the unique abilities of dentists to provide enhanced, accessible care by taking away routine parts of dentists’ work.

Artificial intelligence has contributed substantially to different subfields of dentistry over the past decade. This review discusses numerous applications of AI in periodontology, endodontics, orthodontics, restorative dentistry, and oral pathology including studies incorporating deep learning to effectively diagnose dental disease and plan treatments. However, the current evidence of AI is still sparse due to limitations in terms of dataset availability, size, performance, and generalizability. This study highlights the limitations of the relevant studies and provides future directions which may have a positive impact on the future application of AI-based networks within dentistry. This review indicates that more flexible systems are needed to reach human-level performance and increase the reliability of AI-based clinical decision support systems in dental practice.

## Figures and Tables

**Table 1:** Search strategy conducted in different databases.

Search #	Topic	Terms
1	Artificial intelligence	artificial intelligence“[MeSH Terms] OR (“artificial” [All Fields] AND “intelligence”[All Fields]) OR “artificial intelligence”[All Fields] OR (“machine learning”[MeSH Terms] OR “machine learning”[All Fields]) OR (“deep learning”[MeSH Terms] OR (“deep”[All Fields] AND “learning”[All Fields]) OR “deep learning”[All Fields]) OR (“decision support systems, clinical”[MeSH Terms] OR “clinical decision support systems”[All Fields] OR (“fuzzy logic”[MeSH Terms] OR “fuzzy logic”[All Fields])
2	Dentistry	“periodontics” OR “endodontics” OR pathology OR “dental health services”[All Fields] OR “dental”[All Fields] OR “dentally”[All Fields] OR “dentals”[All Fields]) AND (“surgery”[MeSH Subheading] OR “surgery”[All Fields] OR “surgical procedures, operative”[MeSH Terms] OR (“surgical”[All Fields] AND “procedures”[All Fields] AND “operative”[All Fields]) OR “maxillofacial”[All Fields] OR (“craniofacial“ [All Fields] OR (“restorative dent”[Journal] OR (“restorative”[All Fields] AND “dentistry”[All Fields]) OR “restorative dentistry”[All Fields]) OR (“dental implants”[MeSH Terms] OR (“dental”[All Fields] AND “implants”[All Fields]) OR “dental implants”[All Fields])
3		Search #1 and #2

**Table 2:** Summary of input features used for different diagnostic tasks.

AI Technique	Diagnostic Tasks	Target Problem	Input Features
ANN as CDSS	Disease Diagnosis (Classification, Segmentation, Localization)	tooth decay detection [19], vertical root fracture [20], Cervical vertebra maturation degree [21,22]	Intraoral radiographs [19,20], cephalograms [21], orthodontic scans [22]
	Risk Assessment	Bone loss assessment [23], periodontitis risk assessment [9]	Medical records [9,23]
	Treatment Planning and prognosis	Surgery type and extraction decision [24], tooth extraction therapy [25], occlusal and dental caries [26], temporomandibular joint disorders [27,28]	cephalograms [24], electronic health records [25,26,28], magnetic resonance images [27]
Machine Learning	Disease Diagnosis (Classification, Segmentation, Localization)	Plaque segmentation [29], gingivitis identification [30], alveolar bone loss [6], scoring lesions [31], occlusal caries lesions [32], maxillary structure assessment [33], sagittal skeletal patterns [34], facial attractiveness [35], temporomandibular joint disorders [36]	Oral endoscopic images [29], digital photographs [30,35], intraoral radiographs [6], occlusal images [31,32], CBCT images [33], cephalograms [34], magnetic resonance images [36]
	Treatment Planning and Prognosis	Facial and skin mould [37], teeth extraction [38], tooth prognosis [39], dental restorations [40]	Mould images [37], electronic records [38,39], panoramic images [40]
Deep Learning	Disease Diagnosis (Classification, Segmentation, Localization)	Bone loss [5,8,41,42], odontogenic cystic lesion [43], periodontal bone destruction [7,26], bone recession and interradicular radiolucency [44], alveolar bone delineation [45], bone assessment [46], periapical lesions [47,48], vertical root fracture [49], apical lesions [50], interproximal caries [51], distal root assessment [52], proximal and occlusal caries [53], dental implants [54], preserve tooth boundary [23], maxillary sinusitis lesions [42,55,56,57], temporomandibular joint disorders [58], oral cancer [59]	Periapical radiographs [7,41,44,47], panoramic radiographs [5,26,42,43,49,50,54,56,57,58], CBCT images [8,43,48,52,55], high-frequency ultrasound images [45], intraoral ultrasound images [46], bitewing radiographs [51], NILT images [53], digital photographs [23], water’s view radiographs [42], hyperspectral images [59]
	Landmark detection	Cephalometric landmarks [60,61]	cephalograms [60,61]
	Treatment Planning	Differential orthodontic diagnosis [62]	cephalograms [62]

**Table 3:** Summary of related studies for AI application in periodontology.

AI Application	Author, Year (Ref)	Architecture	Data Modality	Dataset Size Split (Train/Val/Test or Train/Test)	Study Factor	Reference Standard (Ground Truth)	Validation Scheme	Results (Performance Metrics/Values)	Conclusion
Disease segmentation	Li et al., 2022 [29]	CNN	Oral endoscope images	607 images Train: 320 images Test: 287 images 320/287	Plaque segmentation	Dentist	NA	Acc: 0.864 IoU: 0.859	The model is helpful in plaque segmentation on small dataset
	Li et al., 2019 [30]	A method based on contrast limited adaptive histogram (CLAHE), gray-level co-occurrence matrix (GLCM), and extreme machine learning	Digital photographs	93 images Train: 73 images Test: 20 images 73/20	Gingivitis identification	NA	NA	Accuracy: 0.74, Sensitivity: 0.75, Specificity: 0.73, Precision: 0.74	The method is helpful for gingivitis identification
Disease localization	Lin et al., 2015 [6]	Level segmentation based on Bayesian or KNN or SVM classifier	Periapical Radiographs	31 images	Alveolar bone loss	NA	Leave one-out	Mean SD True Positive Fraction (TPF): 0.925, True Positive Fraction (FPF): 0.14	The model localizes bone loss areas with high classification effectiveness
Disease detection	Lee et al., 2022 [41]	VGG+Individual CNN	Periapical Radiographs	1740 images Train: 1218 images, Valid: 417 images, Test: 105 images 1218/417/105	Bone loss	Three dentists	NA	Acc: 0.99, AUC: 0.98	The proposed algorithm is helpful in diagnosing periodontal bone loss
	Krois et al., 2019 [5]	Seven layered deep CNN	Panoramic radiographs	1750 images, Train: 1400 images, Valid: 350 images, 1400/350	Bone loss	Three examiners	Ten-fold	Acc: 0.81	The model shows discrimination ability similar to that of dentists
	Kim et al., 2019 [63]	Deep CNN + Transfer learning	Panoramic radiographs	12,179 images, Train: 11,189 images, Valid: 190 images, Test: 800 images 1189/190/800	Bone loss	Dental clinicians	NA	AUROC: 0.95, F1-score: 0.75, Sensitivity: 0.77, Specificity: 0.95, PPV: 0.73, NPV: 0.96	The model is useful in tooth numbering and achieved detection performance superior to that of dental clinicians
	Lee et al., 2019 [43]	GoogleNet InceptionV3 + Transfer learning	Panoramic radiographs and CBCT images	2126 images including 1140 panoramic and 986 CBCT images Train: 1700 images, Test: 426 images, 1700/426	Odontogenic cyst lesion	NA	NA	Panoramic images: AUC—0.847, Sensitivity—0.882, Specificity—0.77, CBCT images AUC—0. 914, Sensitivity—0.961, Specificity—0.771	The model provides higher diagnostic performance on CBCT images in effectively detecting and diagnosing cystic lesions
Disease classification	Moran et al., 2020 [7]	ResNet Inception	Periapical radiographs	467 images, Train: 415 images, Test: 52 images, 415/52	Periodontal bone destruction	NA	NA	Acc: 0.81, Precision: 0.76, Recall: 0.92, Specificity: 0.71, NPV: 0.90	The inception model classifies regions based on the presence of periodontal bone destruction with encouraging performance
Disease segmentation	Khan et al., 2021 [44]	UNet + DenseNet121	Periapical radiographs	200 images, Train: 160 images, Test: 40 images	Bone recession and inter-radicular radioulency	Three experts	NA	mIoU: 0.501, Dice score: 0.569	Automates the process of detecting the presence and shape of caries
	Zheng et al., 2021 [8]	Automatically constrained dense U-Net	CBCT images	100 images	bone lesion identification	Three reviewers	Four-fold	Dice score for different categories: Background: 0.961, Lesion: 0.709, Material: 0.822, Bone: 0.877, Teeth: 0.801	The model is helpful in detecting the correct shape of the lesion and the bone
	Duong et al., 2019 [45]	UNet	High frequency ultrasound images	35 images, Train: 30 images, Test: 5 images, 30/5	Alveolar bone assessment	Three experts	NA	Dice Coefficient: 0.75, Sensitivity: 0.77, Specificity: 0.99	The method yields a higher performance in delineating alveolar bone as compared to experts
	Nguyen et al., 2020 [46]	U-Net with ResNet34 encoder	Intraoral ultrasound images	1100 images, Train: 700 images, Valid: 200 images, Test: 200 images 700/200/200	Alveolar bone assessment	Three examiners	NA	Dice Coefficient: 0.853, Sensitivity: 0.885, Specificity: 0.998	The model has the potential to detect and segment alveolar bone automatically
Disease diagnosis	Li et al., 2020 [64]	Mask RCNN + novel caliberation method	Panoramic radiographs	298 images, Train: 270 images, Test: 28 images 270/28	Periodontitis prediction	Junior dentist	NA	mAP: 0.826, Dice score: 0.868, F1-score: 0.454, Accuracy: 0.817	The model is useful for diagnosing the severity degrees of periodontitis
	Papantonopoulos et al., 2014 [23]	Multilayer Perceptron ANN	Textual	29 subjects	Aggressive periodontitis	NA	Ten-fold	Accuracy: 0.981	The model provides effective periodontitis classification
	Geetha et al., 2020 [19]	Back propagation Neural Network	Intraoral digital radiographs	105 images	Dental caries detection	NA	Ten-fold	Accuracy: 0.971, FPR: 2.8%, ROC: 0.987	The model is helpful for the detection of tooth decay and is independent of visual errors
Risk assessment	Shankarapillai et al. 2012 [9]	Multilayer Feedforward Propagation	Textual	230 subjects	Periodontitis risk assessment	NA	NA	MSE: 0.132	The model can be used for effective periodontitis risk prediction

**Table 4:** Summary of related studies for AI application in endodontics.

AI Application	Author, Year (Ref)	Architecture	Data Modality	Dataset Size Split (Train/Val/Test or Train/Test)	Study Factor	Reference Standard (Ground Truth)	Validation Scheme	Results (Performance Metrics/Values)	Conclusion
Disease detection and classification	Ghaedi et al., 2014 [31]	Circular Hough Transform Based Segmentation	Intraoral optical occlusal tooth surface images	88 images with blue background	Detect and score caries lesions	International Caries Detection and Assessment System (ICDAS) experts	Ten fold	Accuracy: 0.863, Specificity: 0.983, Sensitivity: 0.83	The automated system was helpful in detecting and score caries lesions.
	Berdouses et al., 2015 [32]	Random Forest	Photographic colored images	103 digital images	Occlusal caries lesions	Two pediatric dentists	Ten-fold	Accuracy: 0.80, Precision: 0.86, Recall: 0.86, F1-score: 0.85, ROC: 0.98	The model was able to provide detection performance similar to that of trained dentists
Disease Detection	Pauwels et al., 2021 [47]	CNN + Transfer Learning	Intraoral radiographs	280 images, Train: 168 images, Test 112 images 168/112	Periapical lesions	3 oral radiologists	Five-fold	Sensitivity: 0.79, Specificity: 0.88, ROC-AUC: 0.86	The study explored the potential of CNN-based assessment of periapical lesions and achieved superior performance compared to human observers
	Fukuda et al., 2020 [49]	DetectNet (CNN)	Panoramic radiographs	300 images, Train: 240 images, Test: 60 images 240/60	Vertical root fracture	Three observers	Five-fold	Recall: 0.75, Precision: 0.93, F1-score: 0.83	The model was useful in identifying teeth with vertical root fracture
	Orhan et al., 2020 [48]	Deep CNN (U-Net)	3D CBCT images	153 images	Periapical lesions	Two Oral and maxillofacial radiologist	NA	Recall: 0.89, Precision: 0.95, F1-score: 0.93	The model was able to detect periapical pathosis with 92.8% reliability
	Ekert et al. 2019 [50]	7 layer feed forward CNN	Panoramic radiographs	85 images, Train: 56 images, Valid: 29 images 56/29	Apical lesions	Six independent and experienced dentists	Ten-fold	Avg AUC: 0.85, Confidence Interval (CI): 95%	A moderately deep CNN was helpful in detecting apical lesions and can be helpful in reducing dentists’ diagnostic efforts
Disease diagnosis	Bayraktar & Ayan, 2022 [51]	Deep CNN (YOLO) pretrained using DarkNet-53	Bitewing radiographs	1000 images, Train: 800 images, Test: 200 images 800/200	Interproximal caries lesions	Two experienced dentists	Hold-out	Accuracy: 0.945, Sensitivity: 0.722, Specificity: 0.981, PPV: 0.865, NPV: 0.954, AUC: 0.871	The study shows promising outcomes for detecting caries in bitewing images achieving an accuracy above 90%
Disease classification	Hiraiwa et al., 2019 [52]	AlexNet GoogleNet	CBCT images and panoramic images	Training image patches: Single root group: 11,472, Extra root group: 11,004, Testing image patches: Single root group:32, Extra root group—32	Assessing number of distal roots of mandibular first molars	Two radiologists	Five-fold	Accuracy: 87.4, Sensitivity: 77.3, Specificity: 97.1, PPV: 96.3, NPV: 81.8, AUC: 0.87	The model achieved detection performance superior to that of dental radiologists in differentiating whether distal root was single or with an extra root
Disease segmentation	Casalegno et al., 2019 [53]	Symmetric Autoencoder with skip connections similar to U-Net and encoding path similar to VGG16	Near Infrared transillumination (TI) images	217 grayscale images of upper and lower molars and premolars, Train: 185 images, Test: 32 images 185/32	Proximal and occlusal caries lesion	Two dentists	Monte Carlo	IoU score: Occlusal—0.49, Proximal—0.49, AUROC: Occlusal—0.83, Proximal—0.85	The proposed system has the potential to support dentists by providing higher throughput in detecting occlusal and proximal lesions
Disease diagnosis	Kositbowornchai et al., 2013 [20]	Probabilistic Neural Network	Intraoral radiographs	200 images (50 sound and 150 vertical root fractures), Train: 120, Test: 80 120/80	Vertical root fracture detection	N/A	Three-fold	Sensitivity: 0.98, Specificity: 0.905, Accuracy: 0.957	The model was helpful in diagnosing vertical root fractures using intraoral digital radiographs

**Table 5:** Summary of related studies for AI application in orthodontics.

AI Application	Author, Year (Ref)	Architecture	Data Modality	Dataset Size Split (Train/Val/Test or Train/Test)	Study Factor	Reference Standard (Ground Truth)	Validation Scheme	Results (Performance Metrics/Values)	Conclusion
Disease diagnosis	Chen et al., 2020 [33]	ML-based algorithm based on multisource integration framework	CBCT images	36 CBCT images, Train: 30 images, Test: 6 images 30/6	Assess maxillary structure variation	NA	NA	Dice score of maxilla: 0.80	The method is helpful in assessing maxillary structure variation in unilateral canine impaction
	Nino-Sandoval et al., 2016 [34]	Support Vector Machine (SVM)	cephalograms	229 cephalograms	Sagittal (skeletal) patterns	NA	Ten-fold	Accuracy: 0.741	The non-parametric method has the potential to classify skeletal patterns using craniomaxillary variables
	Yu et al., 2014 [35]	Support Vector Regression (SVR)	Colored Photographs	108 images	Facial attractiveness (most attractive to least attractive)	69 Orthodontists	NA	Accuracy: 0.718	The model was helpful in finding close correlation with facial attractiveness from orthodontic photographs
Treatment planning	Riri et al., 2020 [37]	Tree Based Classification	Extraoral intraoral and mould images	1207 total images, Extraoral: 325 images, Intraoral: 812 images, Mould: 70 images	Facial and skin color features	NA	NA	Accuracy: 0.942, Sensitivity: 0.953, Specificity: 0.996, F1-score: 0.926	The automatic approach was helpful in classification of orthodontic images with encouraging classification performance
	Suhail et al., 2020 [38]	Random Forest Ensemble Learning Method	Patient records (medical charts, x-rays, facial photographs)	287 patient records	Decision making for teeth extraction	Five experts	Five-Fold	Accuracy: 0.944	The RF ensemble classifier was helpful in extraction and treatment planning
Landmark detection in cephalograms	Song et al., 2020 [60]	Pretrained ResNet-50 using transfer learning	X-Ray images	400 cephalograms, Train: 150 images, Testing sets: Test set 1—150 images, Test set 2—100 images	Detect cephalometric landmarks	Two experienced doctors	NA	Successful Detection Rate (SDR): Test 1—0.862, Test 2—0.758	The model was able to achieve satisfying SDR in detecting 19 landmarks
	Kim et al., 2020 [65]	Two stage DNN with stacked hourglass network	Dataset 1: 2075 cephalograms, Dataset 2: 400	cephalograms	Detect cephalometric landmarks	Two experts	NA	SDR: Test set 1—0.883, Test set 2—0.77	The fully automated cephalometric analysis algorithm and web application help in the diagnosis of anatomic landmarks
	Gilmour and Ray, 2020 [66]	Pretrained ResNet-50 with foveated pyramid attention algorithm	cephalograms	400 cephalograms	Detect cephalometric landmarks	NA	Four-fold	SDR: Test set 1—0.883, Test set 2—0.77	The multiresolution approach was useful in learning features across all scales and is promising for large images
	Zhong et al., 2019 [67]	Attention guided deep regression model through 2 stage U-Net	cephalograms	300 cephalograms Train: 150, images Test: 150 images 150/150	Detect cephalometric landmarks	Two experienced doctors	Four-fold	SDR: 86.74%	The attention-guided mechanism ensures that smaller searching scopes and high data resolution with minimum information redundancy and the model is generalizable to other landmarks
	Park et al., 2019 [61]	YOLOv3	cephalograms	1311 cephalograms, Train: 1028 images, Test: 283 images 1028/283	Detect cephalometric landmarks	One examiner	NA	SDR: 0.804 Computational time: 0.05 s	The model was effective in identifying 80 landmarks with 5% higher accuracy compared to top benchmarks
Disease diagnosis	Makaremi et al., 2019 [68]	Customized CNN	cephalograms	1870 cephalograms	Cervical vertebra maturation (CVM) degree	Experts	Three-fold	Accuracy: 0.95	The model was helpful in determining the degree of maturation of CVM and has the potential to be implemented in real world scenario
	Yu et al., 2020 [69]	Modified DenseNet pretrained with ImageNet weights	Lateral cephalograms	5890 cephalograms	skeletal classification	Five orthodontic specialists	NA	Sagittal Accuracy: 0.957, Vertical Accuracy: 0.964	The model shows potential in skeletal orthodontic diagnosis using lateral cephalograms
Treatment plannimng	Lee et al., 2020 [62]	Modified AlexNet	cephalograms	333 cephalograms, Train: 220 images, Valid: 73 images, Test: 40 images	Differential orthodontic diagnosis	NA	Four-fold	Accuracy: 0.919, Sensitivity: 0.852, Specificity: 0.973, AUC: 0.969	The study indicates that the DCNNs-based model can be applied for differential diagnosis in orthodontic surgery
Disease diagnosis	Amasya et al., 2020 [21]	ANN	cephalograms	647 cephalograms, Train: 498 images, Test: 149 images 498/149	Cervical vertebra maturation degree	Two independent observers	NA	Accuracy: 0.869, Sagittal Sensitivity: 0.935, Vertical Specificity: 0.945	The model was helpful in CVM staging and cervical vertebral morphology classification
	Kok et al., 2019 [70]	ANN	cephalograms	300 cephalograms	Cervical vertebrae stages	Orthodontists	Five-fold	AUC: CV1—0.99, CV2—0.96, CV3—0.94, CV4—0.90, CV5—0.91, CV6—0.96	Compared with machine learning algorithms, ANN provides the most stable results with 2.17 average rank on hand-wrist radiographs
	Budiman et al., 2013 [22]	ANN	Orthodontic scans	190 scanned dental casts	Shape of arch form	Three orthodonotics	NA	Accuracy: 0.763	ANN has the potential to identify arch forms with encouraging accuracy
Treatment planning and prognosis	Choi et al., 2019 [24]	ANN	cephalogram	316 cephalograms, Train: 136 images, Valid: 68 images, Test: 112 images	Surgery type and extraction decision	One otthodontist	NA	Accuracy Surgery decision: 0.96, Surgery type and extraction decision: 0.91	The model was helpful in diagnosing and making surgery type and extraction decision effectively and can be used as auxiliary reference when clinicians make a decision

**Table 6:** Summary of related studies for AI application in prosthetic/restorative dentistry.

AI Application	Author, Year (Ref)	Architecture	Data Modality	Dataset Size Split (Train/Val/Test or Train/Test)	Study Factor	Reference Standard (Ground Truth)	Validation Scheme	Results (Performance Metrics/Values)	Conclusion
Treatment planning	Lee et al., 2022 [39]	Decision Tree	Electronic records	94 clinical cases	Tooth prognosis	Three prosthodontists	NA	Accuracy: 0.841	The model was helpful in determining tooth prognosis for effective treatment planning
Disease detection	Abdalla-Aslan et al., 2020 [40]	Cubic SVM based algorithm	Panoramic radiographs	83 images	Dental restorations	Experienced practitioners	Five-fold	For detection Sensitivity: 0.94, For classification Sensitivity: 0.98	The model has the potential to detect and classify dental restorations to promote patient’s health
Disease diagnosis	Lee et al. 2020 [54]	Fine Tuned and pretrained Deep CNN	Panoramic and periapical radiographs	10,770 images, Train: 6462 images, Valid: 2154 images, Test: 2154 images 6462/2154/2154	Dental implants	Periodontists	Ten-fold	AUC:0.971	The model was helpful in the identification and classification of dental implants with performance similar to that of periodontist
	Takahashi et al., 2021 [71]	Deep CNN (ResNet152) pretrained with ImageNet weights	Oral photographs	1,184 images, maxilla: 748 images, mandible: 436 images	Partially edentulous arches	Clincian	NA	Maxilla: Accuracy: 0.995, Recall: 1.00, Precision: 0.25, AUC: 0.99, Mandible Accuracy: 0.997, Precision: 0.25, Recall: 1.00, AUC: 0.98	The method was helpful in classification of dental arches and can be effective in designing removable partial dentures
Disease segmentation	Xu et al., 2018 [72]	Two Hierarchical CNNs	3D dental images	1200 images, Train: 1000, Valid: 50, Testing: 150 1000/50/150	Preserve teeth boundary	NA	NA	Accuracy: Upper dental model: 0.99, Lower dental model: 0.987	The label-free mesh simplification method helped preserve the teeth boundary information using the 3D dental model.
Treatment Planningand Prognosis	Cui et al., 2020 [25]	Triple Classification algorithm (Extreme Gradient Boost (XGBoost))	Electronic health records	4135 records	Tooth extraction therapy	Two prosthodontists	Five-fold	Binary Classification Accuracy: 0.962, Precision: 0.865, Recall: 0.830, Triple Classification Accuracy: 0.924, Precision: 0.879, Recall: 0.836	The model was helpful in predicting tooth extracting therapy with performance superior to that of prosthodontists
	Javed et al. 2020 [26]	ANN	Electronic records	45 records of children	Occlusal caries lesions	NA	Leave one out	Regression co-efficient: 0.99	The model was helpful in occlusal dentinal caries lesions and the study proposes an iOS app for meticulous prediction of caries

**Table 7:** Summary of related studies for AI application in oral pathology.

AI Application	Author, Year (Ref)	Architecture	Data Modality	Dataset Size Split (Train/Val/Test or Train/Test)	Study Factor	Reference Standard (Ground Truth)	Validation Scheme	Results (Performance Metrics/Values)	Conclusion
Disease diagnosis	Orhan et al., 2021 [36]	ML (KNN and Random Forest (RF))	Magnetic Resonance Imaging	Temporomandibular disorders	Pathologists	NA	NA	Accuracy: Condylar changes—0.77, Disk displacement—0.74	The model was found to be optimal in predicting temporomandibular disorders
	Hung et al., 2022 [55]	Three step CNN based on V-Net and SVR	CBCT images	445 images, Train: 311 images, Valid: 62 images, Test: 249 images 311/62/249	Maxillary sinusitis	NA	NA	AUC: Mucosal thickening—0.91, Mucous retention cyst—0.84	The model helped detect and segment mucosal thickening and mucosal retention cyst using low-dosed CBCT scans
	Kuwana et al., 2021 [56]	CNN (DetectNet)	Panoramic radiographs	1174 images	Maxillary sinus lesions	NA	NA	Maxillary sinusitis: Accuracy—0.90–0.91, Sensitivity—0.81–0.85, Specificity—0.91–0.96, Maxillary sinus cysts: Accuracy—0.97–1.00, Sensitivity—0.80–1.00, Specificity—1.00	The model was helpful in detecting maxillary sinus lesions
	Choi et al., 2021 [58]	CNN (ResNet)	Panoramic radiographs	1,189 images, Training: 951 images, Testing: 238 images 951/238	Temporomandibular joint disorders (TMJ) osteoarthritis	Oral and maxillofacial radiologist (OMFR)	Five-fold	Temporal: AUC - 0.93, Geographical external: AUC—0.88	The model achieved significantly higher diagnostic performance compared to that of radiologists
	Kim et al., 2019 [42]	CNN	Water’s view radiographs	200 images	Maxillary sinusitis	Five radiologists	NA	Accuracy: Upper dental model: 0.99, Lower dental model: 0.987	The label free mesh simplification method was helpful in preserving the teeth boundary information using 3D dental model
	Murata et al., 2019 [57]	AlexNet CNN	Panoramic radiographs	120 images	Maxillary sinusitis	Two radiologists, Two dentists	NA	Accuracy: 0.875, Sensitivity: 0.867, Specificity: 0.883	The model shows diagnostic performance similar to the radiologists and superior to the resident dentists
	Jeyaraj et al., 2019 [59]	Partitioned CNN (GoogleNet Inception V3)	Hyperspectral images	600 images	Oral Cancer	Expert oncologist	Seven-fold	Benign tissue Accuracy—0.914, Malign tissue Accuracy—0.945	The model helped predict cancerous or benign tumor and has the potential to be applied as a workbench for automated classification
Disease prognosis	Iwasaki et al. 2015 [27]	Bayesian Belief Network (BNN)	Magnetic Resonance Imaging	590 images	Temporomandibular joint disorders (TMJ)	NA	Ten-Fold	Accuracy: 0.99	The model has the potential to determine the progression of TMD in terms of bone changes, disc displacement and bony space and disc affect with encouraging diagnostic performance
	Bas et al., 2012 [28]	Back Propagation ANN	Electronic records	219 records	Clinical symptoms (Temporomandibular joint disorders (TMJ))	Experienced oral and maxillofacial surgeon	NA	Unilateral with and without reduction: Sensitivity—0.80 & 0.95, Specificity—0.69 & 0.91	The model was helpful in diagnosing the preliminary subtypes of TMJ and can be useful in the decision-making process

**Table 8:** Limitations of relevant studies employing AI for disease diagnosis.

Diagnostic Task	Target Problem	Author, Year (Ref.)	Limitations
Disease diagnosis	bone recession and interradicular radiolucency	Khan et al., 2021 [44]	Limited in terms of size
	temporomandibular joint disorders	Orhan et al., 2021 [36]	Further study is required for identification for the reduction state.
		Choi et al., 2021 [58]	The dataset size is rather small and more images are required to improve performance
	maxillary sinusitis lesions	Hung et al., 2022 [55]	The performance can be further improved
		Kuwana et al., 2022 [56]	Dataset is small in terms of size. The study also did not include post-operative maxillary sinuses
		Kim et al., 2019 [42]	Dataset is limited in terms of size and only includes maxillary sinus using water view radiographs
		Murata et al., 2019 [57]	Limited dataset in terms of size
	alveolar bone delineation	Duong et al., 2019 [45]	Limited dataset in terms of size
	bone assessment	Nguyen et al., 2020 [46]	The model is restricted to detection and segmentation in buccal surfaces
	periapical lesions	Orhan et al., 2020 [48]	The model can be influenced with variations i.e., presence of endo-perio lesions or other periodontal defects
	bone loss	Lee et al., 2022 [41]	The model is not able to detect vertical defect depth and angulation. The diagnosis also relies on the reliability of examiners
		Kim et al., 2019 [63]	Low resolution for the individual tooth in panoramic images as it captures a wide field of view
		Krois et al., 2019 [5]	Limited dataset in terms of size
		Zheng et al., 2021 [8]	The approach is not applicable on unlabeled data
		Papantonopoulos et al., 2014 [23]	The model is not flexible enough to capture nonlinearities in data
	oral cancer	Jeyaraj et al., 2019 [59]	Dataset is limited in terms of size
	odontogenic cystic lesion	Lee et al., 2019 [62]	Limited dataset in terms of size
	periodontal bone destruction	Moran et al., 2020 [7]	Poor performance in classifying healthy regions due to small dataset
	vertical root fracture	Fukuda et al., 2020 [49]	The model is trained on panoramic radiographs with clear vertical root fracture (VRF) lines which impacts the performance
	apical lesions	Ekert et al., 2019 [50]	Manually cropped image segments were used for training The sensitivity should be improved before clinical use
	interproximal caries	Bayraktar & Ayan, 2022 [51]	Dataset is limited in terms of size. Carious lesions were not classifiedas enamel caries or dental caries
	distal root assessment	Hiraiwa et al., 2019 [52]	Image patches are created by manual segmentation which is time-consuming.
	proximal and occlusal caries	Casalegno et al., 2019 [53]	Limited dataset in terms of size and ground truth labels
	dental implants	Lee et al., 2020 [43]	The proposed study only includes three types of dental implants which limit its practical use
	preserve tooth boundary	Takahashi et al., 2021 [71]	Limited dataset in terms of size of maxilla set
Disease diagnosis	tooth decay detection	Geetha et al., 2018 [19]	Small dataset The model does not provide classification based on caries depth
	alveolar bone loss detection	Lin et al. 2015 [6]	Poor performance on unevenly illuminated images
	occlusal caries lesion detection	Berdouses et al., 2015 [32]	The accuracy can be improved further
	sagittal skeletal patterns identification	Nino-Sandoval et al., 2016 [34]	Limited class discrimination
	vertical root fracture identification	Kositbowornchai et al., 2013 [20]	The proposed model is not applicable on other root fractures in clinical practice
	scoring lesions	Ghaedi et al. 2014 [31]	Small dataset Unbalanced number of images, histological verification of the diagnosis was not performed
	cervical vertebra maturation degree	Budiman, 2013 [22]	There is an overlap in data distribution that affects the models’ performance
	facial attractiveness	Yu et al., 2014 [35]	The model prediction is limited to certain angles and ratios
	plaque segmentation	Li et al., 2022 [29]	The model is not generalizable to other caries areas
	detect vertical vertebra maturation degree	Amasya et al. 2020 [21]	Hand-wrist radiographs are not considered
	maxillary structure assessment	Chen et al., 2020 [33]	The dataset is limited in terms of size

**Table 9:** Limitations of relevant studies employing AI for different treatment planning, landmark detection, and risk assessment.

Diagnostic Task	Target Problem	Author, Year (Ref.)	Limitations
Treatment planning and prognosis	surgery type and extraction decision	Choi et al., 2019 [24]	The study does not include skeletal asymmetry cases
	tooth extraction therapy	Cui et al., 2020 [24]	Other factors such as adjacent teeth, subsequent treatments are not considered in this study
	orthodontic diagnosis	Lee et al., 2020 [62]	Limited dataset in terms of size
Landmark detection	cephalometric landmark identification	Song et al., 2020 [60]	The computational time still needs further improvement
		Yu et al., 2020 [69]	Lacks in terms of performance
		Li et al., 2020 [64]	Small dataset Poor performance on radiographs with severe periodontitis with abnormal shape of teeth
		Kim et al., 2020 [65]	Not applicable to all landmarks in clinical situations
		Gilmour and Ray, 2020 [66]	The bottleneck is in terms of storage as each iteration only loads small glimpse of image
		Zhong et al., 2019 [67]	Limited in terms of computational capabilities
		Park et al. 2019 [61]	Intra/inter-examiner reliability statistics and reproducibility comparisons are required
Risk Assessment	periodontitis risk assessment	Shankarapillai et al., 2012 [9]	Other metrics, i.e., accuracy have not been reported

## Data Availability

Data is available from the authors on request.
